# Intracellular Helix-Loop-Helix Domain Modulates Inactivation Kinetics of Mammalian TRPV5 and TRPV6 Channels

**DOI:** 10.3390/ijms24054470

**Published:** 2023-02-24

**Authors:** Lisandra Flores-Aldama, Daniel Bustos, Deny Cabezas-Bratesco, Wendy Gonzalez, Sebastian E. Brauchi

**Affiliations:** 1 Instituto de Fisiología, Facultad de Medicina, Universidad Austral de Chile, Valdivia 5110566, Chile; 2 Department of Neuroscience, University of Wisconsin School of Medicine and Public Health, 1111 Highland Ave. #5505, Madison, WI 53705, USA; 3 Centro de Investigación de Estudios Avanzados del Maule (CIEAM), Vicerrectoría de Investigación y Postgrado, Universidad Católica del Maule, Talca 3460000, Chile; 4 Laboratorio de Bioinformática y Química Computacional (LBQC), Departamento de Medicina Traslacional, Facultad de Medicina, Universidad Católica del Maule, Talca 3460000, Chile; 5 Center for Bioinformatics and Molecular Simulations (CBSM), University of Talca, Talca 3460000, Chile; 6 Millennium Nucleus of Ion Channel-associated Diseases (MiNICAD), Valdivia 5110566, Chile

**Keywords:** TRP channels, inactivation, TRPV5, TRPV6, molecular evolution

## Abstract

TRPV5 and TRPV6 are calcium-selective ion channels expressed at the apical membrane of epithelial cells. Important for systemic calcium (Ca^2+^) homeostasis, these channels are considered gatekeepers of this cation transcellular transport. Intracellular Ca^2+^ exerts a negative control over the activity of these channels by promoting inactivation. TRPV5 and TRPV6 inactivation has been divided into fast and slow phases based on their kinetics. While slow inactivation is common to both channels, fast inactivation is characteristic of TRPV6. It has been proposed that the fast phase depends on Ca^2+^ binding and that the slow phase depends on the binding of the Ca^2+^/Calmodulin complex to the internal gate of the channels. Here, by means of structural analyses, site-directed mutagenesis, electrophysiology, and molecular dynamic simulations, we identified a specific set of amino acids and interactions that determine the inactivation kinetics of mammalian TRPV5 and TRPV6 channels. We propose that the association between the intracellular helix-loop-helix (HLH) domain and the TRP domain helix (TDh) favors the faster inactivation kinetics observed in mammalian TRPV6 channels.

## 1. Introduction

Systemic calcium (Ca^2+^) homeostasis is vital for many physiological processes. Maintaining Ca^2+^ levels within a narrow range in extracellular fluids is regulated by the rates of absorption, storage, and excretion. These processes are mediated by passive and active transport mechanisms [1,2]. Active transcellular transport is a regulated process involving three steps: apical entry of Ca^2+^ into epithelial cells, transport to the basolateral membrane, and extrusion to the bloodstream [1,2]. This cellular mechanism is maintained by a specific set of transporters, pumps, and ion channels [1,2].

TRPV5 and TRPV6 are calcium-selective ion channels belonging to the Transient Receptor Potential (TRP) family [3]. Expressed at the apical membrane of calcium-transporting epithelia, these channels serve as entry channels in the transcellular pathway [1,2,3]. In accordance with this role, their tissue expression pattern correlates with the habitats and physiological requirements of vertebrate animals. In fish, the unique TRPV5/6 homolog is expressed in gills, where the majority of Ca^2+^ absorption occurs, while in terrestrial animals, TRPV5-6 paralogs are mainly expressed in the kidney, playing a crucial role in the cation re-absorption [4]. These changes in the expression pattern probably contributed to adaptation to terrestrial life. In mammals, TRPV5 and TRPV6 expression profile is clearly defined. While TRPV5 is mainly expressed in kidneys, TRPV6 has been identified in multiple organs such as the brain, kidneys, intestine, muscle, testis, and placenta [4,5]. When expressed in kidneys, TRPV6 channels are exposed to highly regulated Ca^2+^ extracellular concentration under physiological conditions. Nevertheless, intestinal TRPV6-expressing epithelial cells are exposed to quick changes in the Ca^2+^ extracellular levels after every meal. Moreover, TRPV6 expression is regulated by dietary Ca^2+^ levels [6,7,8]), and its relevance in intestinal epithelial barrier dysfunction was recently discussed by Mori et al. [9]. This suggests a clear division of their physiological functions and probably regulatory mechanisms.

Single nucleotide polymorphisms in human TRPV5 and TRPV6 have been related to a higher mineral bone density and the development of kidney stones in African descendent populations compared to Caucasians [5,10,11,12]. Moreover, altered expression of TRPV5 and TRPV6 has been related to cancer [13,14,15,16,17], multiple pathologies of bone [18,19,20,21,22] and kidney [20,23,24,25], and impairments in reproductive physiology [26,27,28]. In agreement with their physiological relevance, the expression levels, trafficking, and activity of these channels are highly regulated [1,29,30,31]. An increase in the intracellular Ca^2+^ concentration induces fast and slow types of inactivation in calcium-selective TRP channels TRPV5 and TRPV6 [32]. Slow inactivation is common to both, and this is determined by the binding of the Ca^2+^/Calmodulin complex to the channel’s intracellular region [31,33]. Based on functional and structural data, it has been hypothesized that calmodulin (CaM) blocks the channel’s intracellular gate via a cation–π interaction between a lysine side chain from CaM (Lys116 in rat CaM) and tryptophan residues located at the lower gate of the channels (Trp583 in rabbit TRPV5]. Moreover, structural data revealed a state-dependent interaction between charged residues located at the ankyrin repeat domain 6 (ARD6) and the helix-loop-helix (HLH) domain [31]. In addition to the slow inactivation component, a strong, fast inactivation is observed only in mammalian TRPV6 channels [32,34]. Our phylogenetic and functional studies suggested that calcium-dependent fast inactivation corresponds to an evolutive innovation in vertebrates that is absent in fish and nearly absent in reptiles [4].

Different groups have mapped residues associated with fast inactivation. It has been found that amino acids located at the helix-loop-helix (HLH) domain (residues E288, F292, and S298 in human TRPV5 (hTRPV5) [4] and the intracellular linker between the transmembrane segments 2 and 3 (S2-S3 linker; residues L409, V411, and T412 in human TRPV6 (hTRPV6) [34] form part of the mechanism of inactivation. In this context, our previous data suggest a strong evolutionary correlation between residues located at the HLH domain and the S2-S3 linker, supporting the robust inactivation displayed by mammalian TRPV6 channels [4].

In this study, the sequence conservation of each component of the HLH/S2-S3 linker/TRP domain helix (TDh) inactivation motif was thoroughly examined. It was discovered that scaffold sequences at the HLH and S2-S3 linker contain specific residues responsible for fast inactivation. We found variations in the connectivity network within this putative inactivation motif by carefully examining the three-dimensional structures of mammalian TRPV5 and TRPV6 channels in the apo and Ca^2+^/CaM-bound states. Individual residues responsible for inactivation were revealed via site-directed mutagenesis and patch-clamp electrophysiological recordings. Lastly, molecular dynamics simulations suggest a mechanism where the mammalian TRPV6 fast inactivation is likely dependent on the close proximity between the HLH domain and the TDh.

## 2. Results

### 2.1. The Evolutionary Profiles of the Putative Inactivation Motif

**Figure 1:**
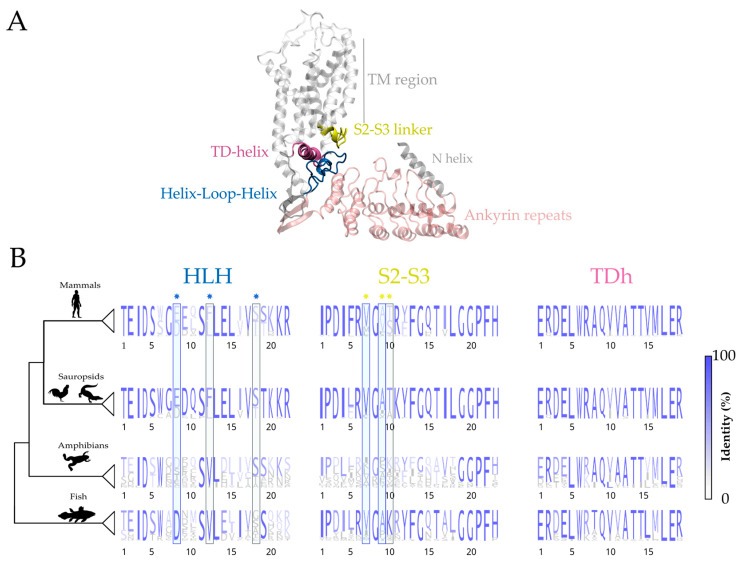
Helix-loop-helix (HLH) domain, S2-S3 linker, and TRP domain helix (TDh) show independent evolution patterns. (**A**) Structural model of one subunit of the human TRPV6 channel (hTRPV6, PDB:7S88). Ankyrin repeats domain (ARD, light pink), HLH domain (blue), S2-S3 linker (yellow), and TDh (mauve) are highlighted. TM region: transmembrane region, N helix: Amino-terminal helix. (**B**) Residue identity percent (in size and blue shades) for the components of the HLH/S2-S3 linker/TDh inactivation motif. Residues at the HLH domain (blue asterisks) and S2-S3 linker (yellow asterisks) previously shown as critical for TRPV6 fast inactivation are highlighted. Shadows represent functionally relevant amino acids not conserved (gray) and conserved (blue) in amphibians and/or fish compared to sauropsid and mammalian homologs. Multiple sequence alignment in Supplementary Figure S1 was used to calculate the percentage of amino acid identity.

We first explored the elements of the HLH/S2-S3 linker/TDh motif by studying their primary sequences (Figure 1). The HLH and the S2-S3 linker display higher sequence identity in mammals and sauropsids and are evidently more variable in amphibians and fish (Figure 1B). In contrast, the consensus sequence corresponding to the TDh is relatively conserved in all examined groups (Figure 1B). This suggests independent evolutionary transitions for the different structural pieces forming the motif.

In a previous study, we reported independent duplication events occurring in mammals and sauropsids [4]. In both cases, this duplication originates an evolutionary innovation in the form of a fast-inactivating channel paralog. Here, our sequence analysis revealed that in mammals and sauropsids, the HLH domain has a scaffold of well-conserved amino acids around the residues shown to be critical for fast inactivation (Figure 1B, blue asterisks, D^8^L^12^T^18^ amino acid triad in mammalian TRPV6]. Interestingly, such a conserved scaffold is partially absent in amphibians and fish, whose Ca^2+^-selective TRP channels lack fast inactivation [4]. Additionally, critical residues that are a fingerprint for fast-inactivating mammalian TRPV6 channels (Figure 1B, L^12^T^18^, gray shadows) are not conserved in amphibians and fish, suggesting that these TRPV5-6 homologs have never evolved towards a fast-inactivating phenotype (Figure 1B).

On the other hand, a similar but less strong pattern is detectable at the S2-S3 linker. Here, mammals and sauropsids have a more conserved set of amino acids around the residues defining the phenotype of fast inactivation (Figure 1B, yellow asterisks). Moreover, a threonine residue at position 10 (gray shadow) is highly conserved in fast-inactivating mammalian TRPV6 channels, and it is substituted by well-conserved positively charged residues in amphibian and fish TRPV5/6. However, two S2-S3 linker amino acids previously associated with inactivation kinetics in mammalian TRPV5 channels (V^7^A^9^ blue shadows) are conserved in fish. All this suggests that scaffolds of well-conserved residues at the HLH and S2-S3 linker domains are required, although not sufficient to support fast inactivation, and that the necessary fit between these two elements (HLH and S2-S3 linker) was not fully developed until the duplication event occurring at the mammalian ancestor. 

Evolutionary reconstruction of the Transient Receptor Potential Vanilloid (TRPV) subfamily showed that calcium-selective TRPV5-6 and temperature-sensitive TRPV1-4 form independent monophyletic groups sister to each other [4]. Our sequence analysis reveals unique variations in these groups’ primary structures at the regions of interest, where the inclusion of hTRPV1-4 channels caused divergences in our alignment at both the HLH and S2-S3 linker (Supplementary Figure S1, zoomed regions black arrows). The amino acid composition of the S2-S3 linker varied significantly between the analyzed monophyletic groups. In contrast to the mammalian calcium-selective TRP homologs, we found greater similarity between hTRPV1-4 and fish TRPV5/6 when comparing residues at the HLH. Moreover, residues associated with inactivation kinetics in mammalian TRPV5-6 [4] are not conserved in hTRPV1-4 (Supplementary Figure S1, black stars). This finding suggests that similarly to amphibians and fish TRPV5/6, hTRPV1-4 did not evolve towards a fast Ca^2+^-dependent inactivation. 

Taken together, our sequence analyses suggest that molecular evolution associated with fast inactivation in mammalian TRPV5-6 channels was built upon the stabilization of a conserved scaffold sequence surrounding specific residues located at the HLH and the S2-S3 linker. 

### 2.2. The Distance between the HLH and TDh Modulates Fast Inactivation 

In the absence of a detailed inactivation mechanism, we worked under the underlying assumption that both phases of inactivation, fast and slow, converge into a structurally similar fully inactivated state. Accordingly, the apo/lipid-bound condition, defined by the absence of a bound ligand—other than lipids used for protein purification and imaging—was considered a non-inactivated state. Moreover, the Ca^2+^/CaM-bound structures were assumed as fully inactivated channels. To explore structural rearrangements that occurred during inactivation, we compared the available structures of mammalian TRPV5 and TRPV6 in the apo/lipid-bound (PDB IDs for TRPV5: 6DMR, 6O1P; PDB IDs for TRPV6: 6BO8, 6BO9, 6BOB 7K4A, 7S88, 7S89) and Ca^2+^/CaM-bound states (PDB IDs for TRPV5: 6DMW, 6O20; PDB IDs for TRPV6: 6E2F, 6E2G).

**Figure 2:**
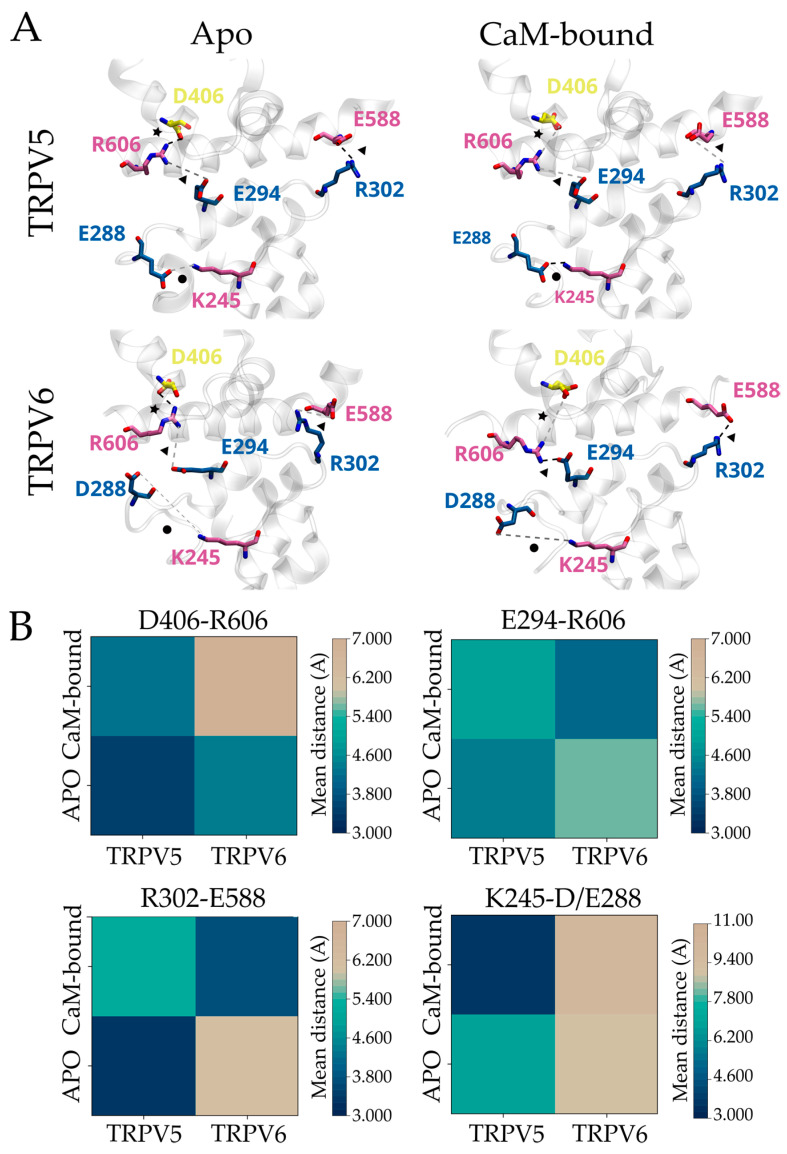
Evolutionary amino acid substitutions modulate the three-dimensional conformation of the HLH/S2-S3 linker/TDh inactivation motif in mammalian TRPV5 and TRPV6. (**A**) Representative structural arrangement of the three-dimensional inactivation motif of mammalian TRPV5 and TRPV6 in the apo/lipid-bound and Ca^2+/^CaM-bound conformations (PDBs; TRPV5 apo: 6DMR, TRPV5 Ca^2+^/CaM-bound: 6DMW, TRPV6 apo: 7S88, TRPV6 Ca^2+^/CaM bound: 6E2F). Residues located at HLH (blue), S2-S3 linker (yellow), TDh (mauve), and ARD6 (pink) contributing to interdomain interactions are highlighted. Interactions between HLH–TDh (triangle), HLH–ARD6 (circle), and TDh/S2-S3 linker (star) are highlighted. (**B**) Heat maps representing the average distance between the pairs of residues in panel **A**. Bars on the right of each heat map represent the color scale of the mean distance in the analyzed structures.

To evaluate possible interactions within the inactivation motif, we measured the distance between residues located at the different domains (i.e., charged side chains putatively involved in interdomain salt bridges) in the available non-inactivated and fully inactivated TRPV5-6 structures. Average distances from several structural models were obtained for each condition and further used as a proxy to analyze changes in interdomain interactions (Figure 2B). This close inspection revealed a specific set of residues that are in close proximity, suggesting a contribution to interdomain associations (Figure 2A).

In non-inactivating TRPV5 channels, the S2-S3 linker is closer to the TDh when compared to Ca^2+^-inactivating TRPV6 (i.e., the averaged distance between D406 and R606, star in Figure 2A) (Figure 2B). This observation extends to all other interdomain interactions, including the HLH with respect to the TDh (E294-R606 and R302-E588, triangle in Figure 2A), and the ankyrin repeat domain 6 (ARD6) to the HLH (K245-E288, circle in Figure 2A) (Figure 2B). 

In TRPV5, the majority of these interdomain distances increased when the Ca^2+^/CaM complex was bound (Figure 2B). Notably, the distance between ARD6 and HLH (K245-E288) is clearly shorter in the fully inactivated TRPV5 when compared to the apo structure and to any conformation available for TRPV6 (Figure 2B). In contrast, the binding of the Ca^2+^/CaM complex to TRPV6 had a different effect on the interdomain distances. While residues located at the S2-S3 linker were found farther away from the TDh (i.e., D406-R606), we observed that the TDh and the HLH domain remained in close contact (i.e., E294-R606 and R302-E588) (Figure 2B). In the fully inactivated TRPV6 channels, the proximity between the HLH and TDh seems to be stabilized by the interaction between R302-E588 and E294-R606. We reasoned that the latter interaction prevents the closer contact between R606 and D406 (i.e., TDh and S2-S3 linker), observed in Ca^2+^/CaM-bound TRPV5, placing the HLH and TDh close to each other and away from the S2-S3 linker. In all conformations analyzed for TRPV6, the HLH and ARD6 are found apart from each other (Figure 2B).

**Figure 3:**
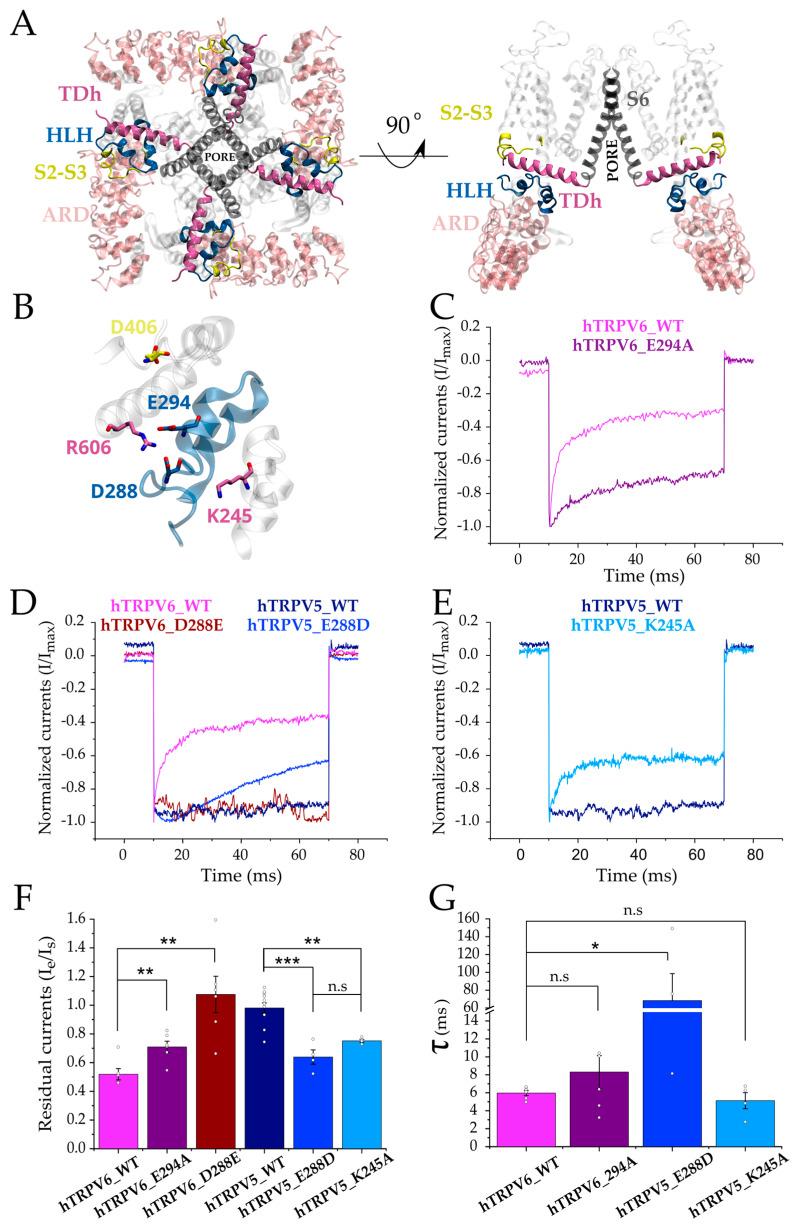
HLH interaction network modulates the inactivation kinetics of hTRPV5 and hTRPV6 ion channels. (**A**) Structure of the tetrameric hTRPV6 ion channel (PDB: 7S88). The structure shows the TDh (mauve) connecting the S6 segment (dark gray) with the HLH (blue) and S2-S3 linker (yellow) domains. ARD in close contact with the HLH is shown in pink. The right panel shows two subunits for easier visualization. (**B**) Representation of residues involved in the differential interdomain interactions of the HLH (in blue) within the inactivation motif of hTRPV5 and hTRPV6 (numbers according to hTRPV6). (**C**) Normalized whole-cell current traces recorded from transiently transfected HEK-293T cells expressing wild-type (WT) hTRPV6 and mutant hTRPV6–E294A channels. (**D**) Normalized whole-cell current traces recorded from transiently transfected HEK-293T cells expressing WT hTRPV5, hTRPV6, and mutant hTRPV6–D288E and hTRPV5–E288D channels. (**E**) Normalized whole-cell current traces recorded from transiently transfected HEK-293T cells expressing WT hTRPV5 and mutant hTRPV5–K245A channels. (**F**) Pooled data comparing the residual currents (defined as the ratio between the currents at the end (Ie) versus the beginning (Is) of the voltage pulse). (**G**) Pooled data for the time constants of inactivation for clones showing a fast current decay. All current traces were recorded with 2 mM extracellular Ca^2+^ and elicited in response to a 60 ms pulse at −160 mV from Vh = 0 mV (Panels **C**,**D**,**E**). Bars represent mean value; errors represent S.E.M, and white dots represent values for each experiment (Panels **F**,**G**). *** represents *p*  =  0.001; ** *p*  =  0.01; * *p* = 0.05; n.s. = not statistically significant.

The TDh runs parallel to the plasma membrane and is directly connected to the channel’s internal gate (Figure 3A). Thus, it is reasonable to think that the TDh drives the inactivation by coupling changes occurring in the landscape of interactions between the HLH and S2-S3 linker to the channel internal gate after binding Ca^2+^/CaM (Figure 3A). We and others have previously reported that the HLH and S2-S3 linker have a critical role in the fast inactivation of mammalian TRPV6 channels [4,34]. Therefore, in both inactivation mechanisms, rearrangements occurring in the association between the TDh to the HLH and S2-S3 linker would modulate inactivation kinetics.

To test this hypothesis directly, we used site-directed mutagenesis and patch-clamp electrophysiology in whole-cell configuration following a 60 ms-negative voltage command, as previously reported by Hoenderop et al. [32]. Following the same protocol, our internal recording solutions contained 10 mM EGTA to keep low levels of intracellular Ca^2+^ concentration, benefiting high-calcium levels at the vicinity of the channels’ internal pore. We first revisited the cation-specificity of the fast inactivation mechanism in hTRPV6 [34]. Electrophysiological recordings in the presence of 10 mM extracellular Ba^2+^ showed an inward current that remained stable during the recording time (Supplementary Figure S2). As expected from previous reports, substituting the extracellular Ba^2+^ with 2 mM Ca^2+^ was sufficient to induce inactivation, demonstrating the strong calcium specificity of the current decay (traces in pink and magenta Supplementary Figure S2). 

To study the relevance of the residues associated with the interdomain associations described above, we first disrupted the interaction between the HLH and TDh in hTRPV6 (E294-R606) by neutralizing the E294 negative charge. Patch-clamp recordings in whole-cell configuration showed that hTRPV6–E294A mutation partially prevents fast inactivation in hTRPV6 when compared to the wild-type (WT) channel (Figure 3C,F,G and Supplementary Figure S3). The interacting E294 and R606 are highly conserved amino acids in TRPV5 and TRPV6, forming part of what we defined as scaffold sequences (Figure 1B positions 14 and 19 in HLH and TDh panels, respectively).

Sequence and structural analyses highlight the amino acid at position 288 (Figure 1B, position 8 in the HLH panel), which is part of the previously described inactivation signature [4]. While in TRPV6, this position is occupied by an aspartate exposed to the solvent, in TRPV5, it is a glutamate side chain that is in close contact with the K245 (located at the ARD6) in both the non-inactivated and fully inactivated states (Figure 2). Even though aspartate and glutamate are negatively charged amino acids, they differ in their side-chain length. This simple variation in length could be the molecular basis that modulates the putative interdomain interactions required for inactivation. To test this idea, we exchanged D288, which is present in hTRPV6 by the glutamate of hTRPV5. Whole-cell patch-clamp recordings showed that the amino acid swapping caused a complete exchange of the fast inactivation phenotype (Figure 3D,F,G, and Supplementary Figure S3). While hTRPV5–E288D showed a rapid current decay after activation, hTRPV6–D288E did not inactivate during the recording time. These results suggest that an interaction between the HLH and the ARD6 (i.e., E288–K245 in TRPV5) might stabilize the open non-inactivated state of the channel. To test this directly, we disrupted the putative electrostatic interaction by eliminating the K245 positive charge (hTRPV5–K245A). The hTRPV5–K245A mutant showed an evident fast inactivation that is absent in the WT counterpart (Figure 3E–G and Supplementary Figure S3). 

Notably, the fast inactivation introduced by mutagenesis in TRPV5 channels was also found specific for Ca^2+^, as Ba^2+^ was unable to induce inactivation. Like hTRPV6 ion channels, hTRPV5–E288D and hTRPV5–K245A only showed a fast current decay after substituting the extracellular Ba^2+^ with Ca^2+^ ions (Supplementary Figure S4).

Altogether, these results strongly suggest that close proximity between the HLH and the C-terminal region of the TDh (i.e., E294-R606) favors a calcium-specific fast inactivation observed in hTRPV6 channels. Moreover, this association is likely modulated by the interaction between critical residues located at the HLH with a positively charged residue located at the ARD6. We reasoned that this HLH–ARD6 interaction (i.e., E288–K245 in hTRPV5) might position the HLH away from the TDh, which favors the contact between the TDh and S2-S3 linker (i.e., R606–D406 in hTRPV5) preventing fast inactivation in TRPV5 channels.

### 2.3. Molecular Dynamics Simulations Suggest That Ca^2+^ Ions Drive the Structural Changes Occurring at the Inactivation Motif 

So far, our analyses suggest that the conformational differences between the apo and Ca^2+^/CaM-bound TRPV5 and TRPV6 channels have a critical role in controlling the kinetics of the fast inactivation phase. Therefore, we reasoned that several of the structural rearrangements observed in the fully inactivated channels must occur during the fast phase of the inactivation process. Direct binding has been suggested as the mechanism by which fast inactivation is initiated by Ca^2+^ ions in a concentration-dependent manner [35]. Furthermore, our experiments with extracellular Ba^2+^ highlight the Ca^2+^ specificity of the inactivation process. 

**Figure 4:**
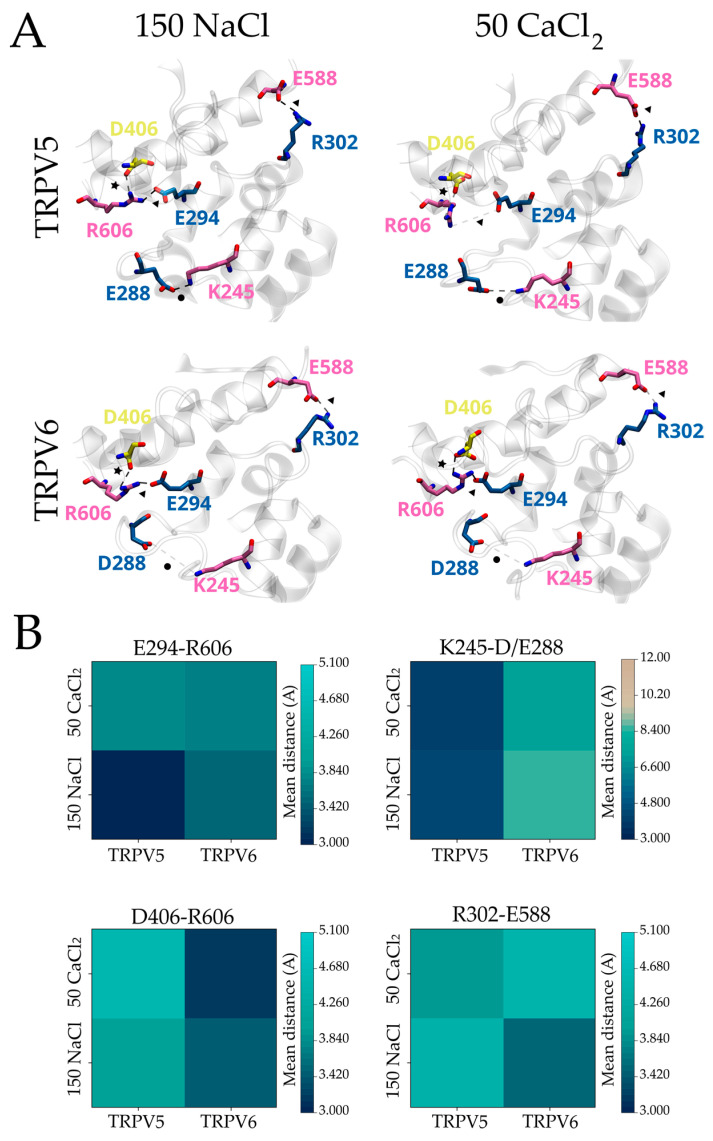
Calcium ions trigger structural changes within the HLH/S2-S3 linker/TDh inactivation motif in mammalian TRPV5 and TRPV6 channels. (**A**) Structural conformation of the three-dimensional HLH/S2-S3 linker/TDh inactivation motif of mammalian TRPV5 and TRPV6 after running 100 ns of fully atomistic molecular dynamics simulations in presence of 150 mM NaCl or 50 mM CaCl_2_ (PDBs; TRPV5: 6DMR, TRPV6: 6BOB). Residues of the HLH (blue), S2-S3 linker (yellow), TDh (mauve), and ARD6 (pink) contributing to interdomain interactions are highlighted, as well as the interactions (HLH/ARD: circle, HLH/TDh: triangle, S2-S3 linker: star). (**B**) Heat maps representing the average distance between the pairs of residues in panel A during the molecular dynamics simulations in presence of 150 mM of NaCl or 50 mM of CaCl_2_. Bars on the right of each heat map represent the color scale of the mean distance during the simulation time.

To explore whether the structural conformation of the HLH domain in the fully inactivated channels associates with Ca^2+^ ions, we performed short (100 ns) fully atomistic molecular dynamics simulations (MDS) (Figure 4 and Supplementary Figure S5) in the presence of 150 mM NaCl or 50 mM CaCl_2_ using apo-state structures (PDBs: 6DMR for TRPV5 and 6BOB for TRPV6). First, we looked for putative Ca^2+^ binding sites during the simulation time (time after reaching stability was determined via RMSD, Supplementary Figure S5). Based on the reported geometry for Ca^2+^ coordination in proteins (four to eight oxygens at an average distance of 2.5 Å or less [36]), we identified the binding of Ca^2+^ ions to four sites located in the ankyrin repeat domains (Supplementary Figure S6). This suggests that direct binding of Ca^2+^ ions to the channel’s intracellular domains might be possible at these putative sites. On the other hand, direct calcium binding to the inactivation motif was observed only when calcium was elevated to 150 mM in the simulations, discouraging the idea of direct calcium binding to the region (data not shown). 

When analyzing the structural rearrangements observed in the interaction network (Figure 4) after calcium is bound, we identified conformational changes similar to those described in the structural analysis. In TRPV6, Ca^2+^ binding decreased the mean distance between the HLH and the C-terminal portion of the TDh (i.e., E294-R606; Figure 4A, triangle) when compared to TRPV5 (Figure 4B). Moreover, while Ca^2+^ binding in TRPV5 brings the HLH and ARD6 (Figure 4A, circle) closer, these residues remain apart in TRPV6 (Figure 4). These results suggest that some of the interactions observed in the fully inactivated channel are likely catalyzed by the binding of Ca^2+^ ions during the rapid phase of the inactivation process. Nevertheless, the average distance between residues located at the S2-S3 linker and TDh (i.e., D406–R606) showed an opposite tendency when compared to the available structures (Figure 2 and Figure 4). While Ca^2+^/CaM-bound TRPV5 structures show the S2-S3 linker close to the TDh, our MDS predicts distancing. Similar opposed behavior was observed in the association between the HLH and the N-terminal portion of the TDh (i.e., R302–E588) when comparing the effects caused by the binding of Ca^2+^ ions to the Ca^2+^/CaM-bound structures. In TRPV6, the binding of Ca^2+^ resulted in an increased distance relative to the Ca^2+^/CaM bound state.

Therefore, we hypothesized that the structural arrangements that are common between the Ca^2+^-bound (from the MDS in the presence of 50 mM CaCl_2_) and the Ca^2+^/CaM-bound (from the fully inactivated structural models) states occur during the fast phase of inactivation, making the structural differences observed rather attributable to conformational changes occurring during the slow phase.

**Figure 5:**
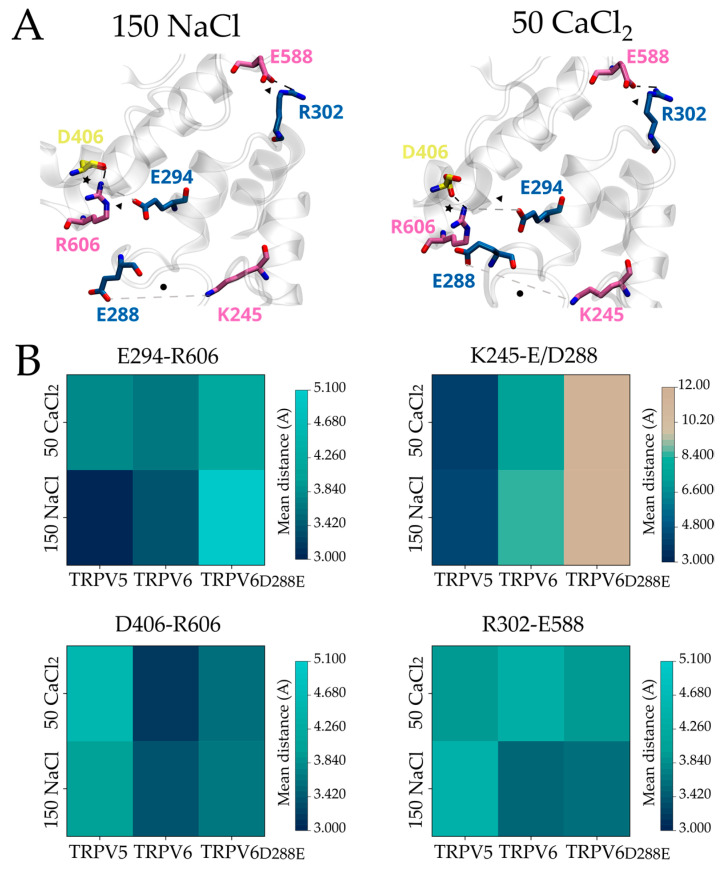
Calcium ions trigger structural changes within the HLH/S2-S3 linker/TDh inactivation motif in mutant TRPV6 channels. (**A**) Structural conformation of the three-dimensional HLH/S2-S3 linker/TDh inactivation motif of TRPV6–D288E after 100 ns of molecular dynamics simulations in presence of Na^+^ or Ca^2+^ ions. Residues of the HLH (blue), S2-S3 linker (yellow), TDh (mauve), and ARD6 (pink) contributing to interdomain interactions are highlighted, as well as the interactions (HLH/ARD: circle, HLH/TDh: triangle, S2-S3 linker: star). (**B**) Heat maps representing the average distance between the pairs of residues in panel A during the simulation time in TRPV5, TRPV6, and TRPV6-D288E. Bars on the right of each heat map represent the color scale of the mean distance during the simulation time.

Finally, we performed MDS using in silico generated non-inactivating TRPV6 mutants. We compared the average distance between residues involved in the interdomain interactions in TRPV6–D288E with the corresponding values obtained for WT TRPV5 and TRPV6 channels in identical simulation conditions (Figure 5 and Supplementary Figure S7). When comparing the behavior of TRPV6–D288E with WT channels in the presence of Ca^2+^, the mutant shows higher similarities to TRPV5 interdomain distances. Relative to TRPV6, the distance between the HLH and the C-terminal region of the TDh (i.e., E294-R606) increases in TRPV5 and TRPV6–E288D, both channels lacking fast inactivation. Similar behavior was observed when comparing the distance between the HLH and the N-terminal portion of the TDh (i.e., R302–E588); in this case, residues are closer in the slow-inactivating channels. However, the distances from the HLH to ARD6 (K245–E288) and the S2-S3 linker to TDh (D406–R606) in TRPV6–D288E showed a tendency similar to the WT TRPV6 interaction network. These might correspond to structural conformations that are intrinsic to mammalian TRPV6 channels and not related to the fast inactivation process.

**Figure 6:**
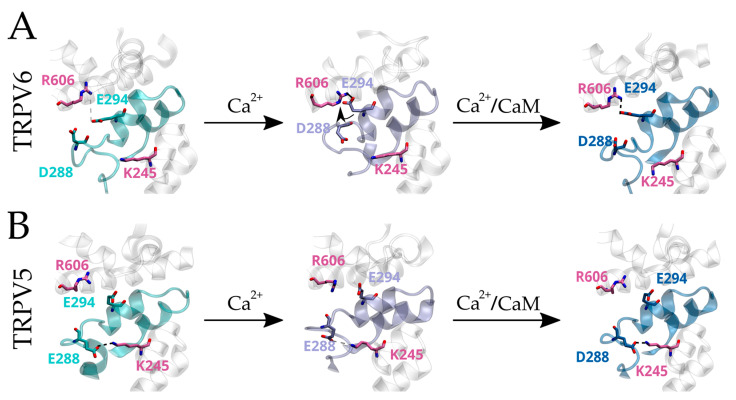
Proposed mechanism of fast inactivation modulation by the HLH in mammalian TRPV6 channels. Conformational changes induced by Ca^2+^ ions and the binding of the Ca^2+^/CaM complex to mammalian TRPV5 and TRPV6 channels. (**A**) Structural rearrangements bring closer the HLH (i.e., E294, blue) to the TDh (i.e., R606, mauve) in TRPV6. (**B**) In mammalian TRPV5, an interaction between the HLH (E288, blue) and the ARD (K245, pink) remains constant in every state, preventing the association between the HLH and TDh.

MDS were also performed for the TRPV6–E294A mutant and the same results were observed (Supplementary Figures S7 and S8). The distance between residues at HLH and TDh (i.e., E294–R606 and R302–E588) shows the same tendency in both TRPV5 and TRPV6–E294A, while the relative positioning between the HLH–ARD6 and S2-S3 linker-TDh of the mutant behaved similarly to WT TRPV6 channels. Therefore, our MDS results suggest that interactions between the HLH and TDh are critical in defining the inactivation phenotype in mammalian TRPV6 channels. Based on our structural and functional analyses, we propose a sequential model for the changes occurring during Ca^2+^-dependent inactivation (Figure 6). These results obtained in silico and their interpretation must be taken carefully and certainly should be studied in more detail. 

## 3. Discussion

### 3.1. Possible Mechanism of Inactivation Modulation by the HLH Domain

Here, we propose that a scaffold of highly conserved amino acids located at the HLH and S2-S3 linker of mammalian TRPV5-6 channels supports the three-dimensional structure required for the correct complementarity between the elements forming the inactivation motif. These scaffold sequences, absent in amphibians and fish TRPV5/6 and hTRPV1-4, were probably present at the common ancestor of both mammalian and sauropsid TRPV5 and TRPV6, while specific amino acid modifications occurred only in one of the duplicated genes in mammals, resulting in fast inactivation as an evolutionary innovation.

Altogether, our evolutionary, structural, and functional analyses suggest that the interactions established between the HLH and the C-terminal portion of the TDh are critical for the modulation of inactivation kinetics in mammalian TRPV5 and TRPV6 channels. In this context, strategies such as trapping the HLH by the ARD6 or stabilizing the interaction between TDh and the S2-S3 linker were important evolutionary traits in non-fast inactivating mammalian TRPV5 channels. We propose that both inactivation mechanisms, fast and slow, likely share some early structural intermediates. 

In our proposed model, once TRPV6 channels open, Ca^2+^ ions permeate through the pore, increasing their local concentration. Elevated local [Ca^2+^] at the channel intracellular microenvironment promotes the interaction between the HLH and C-terminal portion of TDh. Finally, the structural rearrangements occurring at the C-terminal region of the TDh are transmitted to the pore through the same TRP domain helix, whose N-terminal region is physically connected to the channel’s internal gate (Figure 6). In mammalian TRPV5 channels, the interaction between E288 at the HLH with K245 at ARD6 holds the HLH back, preventing its interaction with the TDh, hence, a fast inactivation. The proximity between HLH and TDh in TRPV6 and HLH and ARD6 in TRPV5 is even more evident in the fully inactivated channels suggesting a common ground to both inactivation mechanisms.

### 3.2. Molecular Evolution Defines the Biophysical Properties of TRPV Channels

Our multiple sequence alignment of members of the TRPV subfamily showed a high level of conservation, especially in the TDh, as expected for *bona fide* TRP channels. Nevertheless, some sequence divergences are observed between the sister monophyletic groups TRPV1-4 (thermoTRPV) and TRPV5-6 (calcium-selective TRP) (Supplementary Figure S1). Interestingly some of these differences are in highly relevant functional domains. A significant gap is found in the ARD region, and critical residues for the channel function are different in the HLH domain. Residues located in these regions have been related to the temperature-dependent activation of TRPV1 and TRPV3 [37,38,39,40], while our work suggests the two domains have a significant role in modulating Ca^2+^-dependent fast inactivation, a biophysical property unique to TRPV5 and TRPV6. An additional sequence divergence is found in the S2-S3 linker. To the best of our knowledge, this domain is not involved in modulating the activation by the temperature of TRPV1-4, but we and others have shown this is a key modulator of the fast inactivation in TRPV5-6 [4,34]. Moreover, in other TRP channel subfamilies, the S2-S3 linker binds Ca^2+^, but residues important for such coordination are absent in the TRPV subfamily [41]. Additionally, calcium ions were not noticed to be bound to this intracellular loop, neither in our molecular dynamic simulations nor in any structural data available to date. 

Multiple groups have reported the relationship between the molecular evolution and functional properties of the superfamily of TRP channels, but not much is known about the evolution of TRPV5-6 functional properties. Altogether our results suggest that the molecular evolution within the TRPV subfamily at the intracellular region led to the unique functional properties of the sister groups TRPV1-4 and TRPV5-6. While TRPV1-4 evolved towards a temperature-dependent mechanism of gating, the molecular evolution of TRPV5-6 resulted in slow inactivating channels that further evolved a simple mechanism for fast inactivation in mammalian orthologs.

## 4. Materials and Methods

### 4.1. Sequence Data and Analysis

We retrieved calcium-selective TRP channel sequences in representative species of all major groups of vertebrates. Our sampling included species from mammals, birds, reptiles, amphibians, coelacanths, holostean fish, teleost fish, cartilaginous fish, and cyclostomes (Supplementary Figure S1). Protein sequences were obtained from the Orthologous MAtrix project (OMA) [42]. In cases where the species were not included in the OMA project, we searched in the NCBI database (refseq_genomes, htgs, and wgs) using tbalstn [43] with default settings. Protein sequences were aligned using the FFT-NS-1 strategy from MAFFT v.745 [44]. Human TRPV1, TRPV2, TRPV3, and TRPV4 were used as out groups. Analysis of the sequence conservation was performed using Jalview as well as the figure construction [45].

### 4.2. Molecular Biology, Cell Culture, and Transfection

Open Reading Frames (ORF) codifying to the different channels analyzed (human TRPV5 (hTRPV5) WT, hTRPV5_E288D, hTRPV5_K245A, human TRPV6 (hTRPV6) WT, and hTRPV6_D288E), inserted in a pcDNA3.1(+) vector, were obtained from GenScript Corporation (Nanjing, China). HEK 293T cells were grown in DMEM-F12 medium containing 10% (*v/v*) bovine fetal serum at 37 °C in a humidity-controlled incubator with 5% (*v/v*) CO_2_. HEK 293T cells were transiently co-transfected with the different clones analyzed and peGFP-N1 to allow their identification. Lipofectamine 2000 (Invitrogen) was used for the transfection of the clones according to manufacturer protocol.

### 4.3. Electrophysiology and Solutions

Whole-cell currents were measured with an Axopatch-200B amplifier. We used borosilicate pipettes (o.d. 1.5 mm, i.d. 0.86 mm, Warner Instruments, Hamden, CT) with a resistance of 2 to 4.0 MΩ to form seals with access resistance between 2 and 6 GΩ. Macroscopic currents were recorded in response to a voltage step protocol from zero to −160 mV. The standard extracellular solution (Ringer-Na^+^) contained (in mM) 140 NaCl, 5 KCl, 2 CaCl_2_, 2 MgCl_2_, 8 glucose, and 10 HEPES at pH 7.4 adjusted with NaOH. The Barium solution (Ringer-Ba^2+^) contained (in mM): 128 NaCl, 10 BaCl_2_, 8 KCl, 2 MgCl_2_, 8 Glucose, and 10 HEPES at pH 7.4 adjusted with NaOH. The standard internal (pipette) solution contained (in mM) 105 CsF, 35 NaCl, 10 EGTA, and 10 HEPES at pH 7.4, adjusted with CsOH. Recordings were digitized at 10 KHz and filtered at 5 KHz using a Digitada 1320 (Molecular Devices, LLC, San Jose, CA, USA). All the experiments were performed at room temperature (20 to 25 °C). Inactivation was analyzed in a time window of 60 ms by using Clampfit 10.3 (Molecular Devices, LLC, San Jose, CA, USA). Fast inactivation was assessed by computing the residual currents, defined as the ratio of the current value at the end (last 5 ms) of the negative pulse over the current at the beginning of the voltage pulse (first 5 ms) [4]. The time constant of inactivation was analyzed by fitting the current traces to a single exponential function.

### 4.4. Statistical Analysis

Data are expressed as the mean ± S.E. Overall statistical significance was determined by analysis of variance (ANOVA one way) with a Bonferroni post-test and T-student tests. For all conditions, the average was obtained from at least 4 independent experiments. The outliers were defined using Graphpad QuickCalcs (https://www.graphpad.com/quickcalcs/Grubbs1.cfm, last visited on 26 January 2023) and removed from the analysis.

### 4.5. Structural Analysis of the Available Structures of Mammalian TRPV5-6 Channels

Available three-dimensional structures of mammalian TRPV5 (PDB IDs: 6DMR, 6O1P, 6DMW, 6O20) and TRPV6 (6BO8, 6BO9, 6BOB 7K4A, 7S88, 7S89, 6E2F, 6E2G) were used to study the structural conformation of the HLH/S2-S3 linker/TDh inactivation motif in the apo/lipid- and Ca^2+^/CaM-bound states. The distance between residues putatively involved in interdomain interactions was measured as the distance between the last negatively charged oxygen and positively charged nitrogen in VMD 1.9.3 [46]. Resultant distance values for each structure in each condition were averaged and used as a proxy to study conformational differences.

### 4.6. Molecular Dynamic Simulations (MDS)

Three-dimensional structures of rat TRPV6 (PDB: 6BOB) and rabbit TRPV5 (PDB: 6DMR) were prepared at pH 7.0 and subjected to energy minimization in a vacuum through the Maestro-Schrödinger suite (Schrödinger Release 2018-2: Maestro, Schrödinger, LLC, New York, NY, USA, 2018). The channels were embedded into a pre-equilibrated POPC lipid bilayer in an orthorhombic box with periodic borders, filled with single point charge (SPC) water molecules and an ionic concentration of 150 mM NaCl or 50 mM CaCl_2_. By using these concentrations, our systems have the same ionic strength, and charge screening-driven changes in electrostatic interactions are avoided. Full-atom molecular dynamics simulations of 100 ns were performed, per triplicates, in an NPT ensemble (P = 1 atm, T = 310 K) with the Desmond software and OPLS v.2005 force field [47]. To analyze the interaction network in mutant TRPV6 channels, in silico mutations were introduced by using the plugin residue and loop mutations in the Maestro-Schrödinger Suite. After mutating, the channels were minimized, positioned into systems, and MDS were run as mentioned for the WT channels. Structures were collected every 0.2 ns during the MDS, having 502 frames per simulation. The mean interaction distance between the residues was determined by averaging the measured distance between the last negatively charged oxygen and positively charged nitrogen along the simulation time in VMD 1.9.3 [46]. Root mean square deviation (RMSD) was calculated using VMD 1.9.3 [46].

## Data Availability

Data are contained within the article or Supplementary Material.

## Supplementary Materials

The following supporting information can be downloaded at: https://www.mdpi.com/article/10.3390/ijms24054470/s1.

## References

[1] Van Goor M.K.C., Hoenderop J.G.J., Van Der Wijst J. (2017). TRP channels in calcium homeostasis: From hormonal control to structure-function relationship of TRPV5 and TRPV6. BBA-Mol. Cell Res..

[2] Hoenderop J.G.J., Nilius B., Bindels R.J.M. (2005). Calcium absorption across epithelia. Physiol. Rev..

[3] Van Abel M., Hoenderop J.G.J., Bindels R.J.M. (2005). The epithelial calcium channels TRPV5 and TRPV6: Regulation and implications for disease. Naunyn. Schmiedebergs. Arch. Pharmacol..

[4] Flores-Aldama L., Vandewege M.W., Zavala K., Colenso C.K., Gonzalez W., Brauchi S.E., Opazo J.C. (2020). Evolutionary analyses reveal independent origins of gene repertoires and structural motifs associated to fast inactivation in calcium-selective TRPV channels. Sci. Rep..

[5] Suzuki Y., Chitayat D., Sawada H., Deardorff M.A., Mclaughlin H.M., Begtrup A., Millar K., Harrington J., Chong K., Roifman M. (2018). TRPV6 Variants Interfere with Maternal-Fetal Calcium Transport through the Placenta and Cause Transient Neonatal Hyperparathyroidism. Am. J. Hum. Genet..

[6] Hoenderop J.G.J., Dardenne O., Van Abel M., Van Der Kemp A.W.C.M., Van Os C.H., St-Arnaud R., Bindels R.J.M. (2002). Modulation of renal Ca^2+^ transport protein genes by dietary Ca^2+^ and 1,25-dihydroxyvitamin D_3_ in 25-hydroxyvitamin D_3_-1α-hydroxylase knockout mice. FASEB J..

[7] Song Y., Kato S., Fleet J.C. (2003). Vitamin D receptor (VDR) knockout mice reveal VDR-independent regulation of intestinal calcium absorption and ECaC2 and calbindin D9k mRNA. J. Nutr..

[8] Van Cromphaut S.J., Dewerchin M., Hoenderop J.G., Stockmans I., Van Herck E., Kato S., Bindels R.J., Collen D., Carmeliet P., Bouillon R. (2001). Duodenal calcium absorption in vitamin D receptor-knockout mice: Functional and molecular aspects. Proc. Natl. Acad. Sci. USA.

[9] Mori Y., Omori M., Nakao A. (2022). Vital but vulnerable: Human TRPV6 is a trade-off of powerful Ca2+ uptake and susceptibility to epithelial barrier dysfunction. Cell Calcium.

[10] Akey J.M., Swanson W.J., Madeoy J., Eberle M., Shriver M.D. (2006). TRPV6 exhibits unusual patterns of polymorphism and divergence in worldwide populations. Hum. Mol. Genet..

[11] Renkema K.Y., Lee K., Topala C.N., Goossens M., Houillier P., Bindels J., Hoenderop J.G. (2009). TRPV5 gene polymorphisms in renal hypercalciuria. Nephrol. Dial. Transplant..

[12] Wang L., Holmes R.P., Peng J. (2016). Bin Molecular Modeling of the Structural and Dynamical Changes in Calcium Channel TRPV5 Induced by the African-Specific A563T Variation. Biochemistry.

[13] Chen Y., Liu X., Zhang F., Liao S., He X., Zhuo D., Huang H., Wu Y. (2018). Vitamin D receptor suppresses proliferation and metastasis in renal cell carcinoma cell lines via regulating the expression of the epithelial Ca^2+^ channel TRPV5. PLoS ONE.

[14] Bolanz K.A., Hediger M.A., Landowski C.P. (2008). The role of TRPV6 in breast carcinogenesis. Mol. Cancer Ther..

[15] Song H., Dong M., Zhou J., Sheng W., Li X., Gao W. (2018). Expression and prognostic significance of TRPV6 in the development and progression of pancreatic cancer. Oncol. Rep..

[16] Sun F., Xiao L., Jang X., Xiong Y., Li Q., Yue X., Wei Y.-J., Wei Y.-X., Ma Y.-L., Yu Y.-H. (2016). TRPV6 is a prognostic marker in early-stage cervical squamous cell carcinoma. Tumor. Biol..

[17] Xue H., Wang Y., Maccormack T.J., Lutes T., Rice C., Davey M., Dugourd D., Ilenchuk T.T., Stewart J.M. (2018). Inhibition of Transient Receptor Potential Vanilloid 6 channel, elevated in human ovarian cancers, reduces tumour growth in a xenograft model. J. Cancer.

[18] Wei Y., Jin Z., Zhan H., Piao S., Lu J., Bai L. (2018). The Transient Receptor Potential Channel, Vanilloid 5, Induces Chondrocyte Apoptosis via Ca^2+^ CaMKII-Dependent MAPK and Akt/mTOR Pathways in a Rat Osteoarthritis Model. Cell. Physiol. Biochem..

[19] Chen F., Ni B., Yang Y.O., Ye T., Chen A. (2014). Knockout of TRPV6 causes osteopenia in mice by increasing osteoclastic differentiation and activity. Cell. Physiol. Biochem..

[20] Hoenderop J., Van Leeuwen J.P.T.M., Van Der Eerden B.C.J., Kersten F.F.J., Van Der Kemp A.W.C.M., Mérillat A.M., Waarsing J.H., Rossier B.C., Vallon V., Hummler E. (2003). Renal Ca^2+^ wasting, hyperabsorption, and reduced bone thickness in mice lacking TRPV5. J. Clin. Investig..

[21] Song T., Ma J., Guo L., Yang P., Zhou X., Ye T. (2017). Regulation of chondrocyte functions by transient receptor potential cation channel V6 in osteorarthritis. J. Cell. Physiol..

[22] Song T., Lin T., Ma J., Guo L., Zhang L., Zhou X., Ye T. (2018). Regulation of TRPV5 transcription and expression by E2/ER α signalling contributes to inhibition of osteoclastogenesis. J. Cell. Mol. Med..

[23] Almaimani R.A., Almasmoum H., Ghaitg M.M., El-boshy M., Idris S., Ahmad J., Abdelghany H., BaSalamah M., Mahbub A., Bassem R. (2018). Enhanced remedial effects for vitamin D3 and Calcium co-supplementation against pre-existing lead nephrotoxicity in mice: The roles of renal calcium homeostatic molecules Running. BBA-Mol. Basis Dis..

[24] Liu J., Zhang L., Feng L., Xu M., Gao Y., Zhou P., Zhengmin Y., Zhu B., An Y., Zhang H. (2019). Association between single nucleotide polymorphism (rs4252424) in TRPV5 calcium channel gene and lead poisoning in Chinese workers. Mol. Genet. Genom. Med..

[25] Zeng T., Duan X., Zhu W., Liu Y., Wu W., Zeng G. (2018). Re: saRNA-Mediated Activation of TRPV5 Reduces Renal Calcium Oxalate Deposition in Rat via Decreasing Urinary Calcium Excretion Re: Effectiveness of Treatment Modalities on Kidney Stone Recurrence. J. Urol..

[26] Fecher-Trost C., Lux F., Busch K., Raza A., Winter M., Hielscher F., Belkacemi T., van der Eerden B., Boehm U., Freichel M. (2019). Maternal Transient Receptor Potential Vanilloid 6 (Trpv6) Is Involved In Offspring Bone Development. J. Bone Min. Res..

[27] Tran D.N., Jung E., Ahn C., Lee J., Yoo Y. (2018). Effects of Bisphenol A and 4- tert -Octylphenol on Embryo Implantation Failure in Mouse. Int. J. Environ. Res. Public Health.

[28] Kumar S., Singh O., Singh U., Goswami C., Singru P.S. (2018). Transient receptor potential vanilloid 1-6 (TRPV1-6) gene expression in the mouse brain during estrous cycle. Brain Res..

[29] Lee J., Cha S.-K., Sun T.-J., Huang C.-L. (2005). PIP2 activates TRPV5 and releases its inhibition by intracellular Mg^2+^. J. Gen. Physiol..

[30] Lambers T.T., Oancea E., de Groot T., Topala C.N., Hoenderop J.G., Bindels R.J. (2007). Extracellular pH dynamically controls cell surface delivery of functional TRPV5 channels. Mol. Cell. Biol..

[31] Hughes T.E.T., Pumroy R.A., Yazici A.T., Kasimova M.A., Fluck E.C., Huynh K.W., Samanta A., Molugu S.K., Zhou Z.H., Carnevale V. (2018). Structural insights on TRPV5 gating by endogenous modulators. Nat. Commun..

[32] Hoenderop J.G., Vennekens R., Müller D., Prenen J., Droogmans G., Bindels R.J., Nilius B. (2001). Function and expression of the epithelial Ca^2+^ channel family: Comparison of mammalian ECaC1 and 2. J. Physiol..

[33] Singh A.K., Mcgoldrick L.L., Twomey E.C., Sobolevsky A.I. (2018). Mechanism of calmodulin inactivation of the calcium-selective TRP channel TRPV6. Sci. Adv..

[34] Nilius B., Prenen J., Hoenderop J.G.J., Vennekens R., Hoefs S., Weidema A.F., Droogmans G., Bindels J.M. Fast and Slow Inactivation Kinetics of the Ca2+ Channels ECaC1 and ECaC2(TRPV5 and TRPV6). 2002, 277, 30852–30858. 277.

[35] Nilius B., Prenen J., Vennekens R., Hoenderop J.G., Bindels R.J., Droogmans G. (2001). Modulation of the epithelial calcium channel, ECaC, by intracellular Ca^2+^. Cell Calcium.

[36] Clapham D.E. (2007). Calcium Signaling. Cell.

[37] Yao J., Liu B., Qin F. (2011). Modular thermal sensors in temperature-gated transient receptor potential (TRP) channels. Proc. Natl. Acad. Sci. USA.

[38] Saito S., Tominaga M. (2017). Evolutionary tuning of TRPA1 and TRPV1 thermal and chemical sensitivity in vertebrates. Temperature.

[39] Saito S., Fukuta N., Shingai R., Tominaga M. (2011). Evolution of vertebrate transient receptor potential vanilloid 3 channels: Opposite temperature sensitivity between mammals and western clawed frogs. PLoS Genet..

[40] Liu B., Qin F. (2017). Single-residue molecular switch for high-temperature dependence of vanilloid receptor TRPV3. Proc. Natl. Acad. Sci. USA.

[41] Brauchi S.E., Rothberg B.S. (2020). Gating and calcium-sensing mechanisms of TRPA1 channels revealed. Cell Calcium.

[42] Altenhoff A.M., Glover N.M., Train C.-M., Kaleb K., Vesztrocy A.W., Dylus D., De Farias T.M., Zile K., Stevenson C., Long J. (2018). The OMA orthology database in 2018: Retrieving evolutionary relationships among all domains of life through richer web and programmatic interfaces. Nucleic Acids Res..

[43] Altschul S.F., Gish W., Miller W., Myers E.W., Lipman D.J. (1990). Basic Local Alignment Search Tool. J. Mol. Biol..

[44] Katoh K., Standley D.M. (2013). MAFFT multiple sequence alignment software version 7: Improvements in performance and usability. Mol. Biol. Evol..

[45] Waterhouse A.M., Procter J.B., Martin D.M.A., Clamp M., Barton G.J. (2009). Jalview Version 2-A multiple sequence alignment editor and analysis workbench. Bioinformatics.

[46] Humphrey W., Dalke A., Schulten K. (1996). VMD: Visual molecular dynamics. J. Mol. Graph..

[47] Bowers K., Chow E., Xu H., Dror R., Eastwood M., Gregersen B., Klepeis J., Kolossvary I., Moraes M., Sacerdoti F. Scalable Algorithms for Molecular Dynamics Simulations on Commodity Clusters. Proceedings of the 2006 ACM/IEEE Conference on Supercomputing.

