# Supplementary material for: Intracellular Helix-Loop-Helix Domain Modulates Inactivation Kinetics of Mammalian TRPV5 and TRPV6 Channels
Source: Int J Mol Sci. 2023 Feb 24;24(5):4470. doi: 10.3390/ijms24054470 (PMC10003196; doi:10.3390/ijms24054470)
Supplement: Supplementary file 1 [file ijms-24-04470-s001.zip › ijms-2073146-supplementary.pdf]

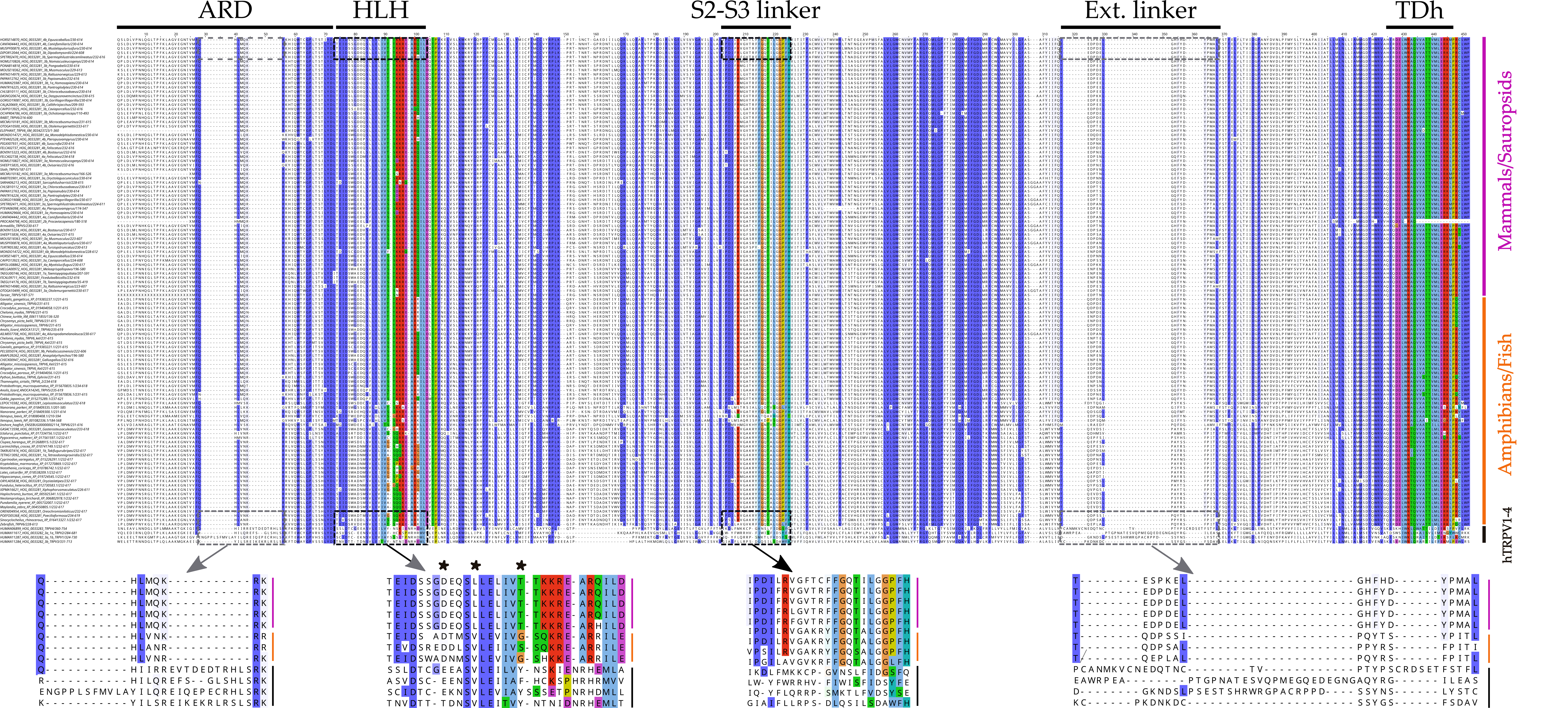
ARD
HLH
S2-S3 linker
Ext. linker
TDh
Mammals/Sauropsids
Amphibians/Fish
hTRPV1-4

SF1

Supplementary Materials Figure S1. Multiple sequence alignment of TRPV5 and TRPV6 in vertebrate representative species. Sequences of hTRPV1-4 were used as an external group. Gene and species are stated to the left, and the amino acid position within the alignment is in the central superior region. Different protein domains are highlighted on the top as: ARD (ankyrin repeat domain), HLH (helix-loop-helix domain), S2-S3 linker (intracellular linker between the transmembrane segments 2 and 3), Ext. linker (extracellular linker known as turret in hTRPV1), TDh (TRP Domain helix). Sequences in zoom highlight the sequence gaps/differences in the HLH, S2-S3 linker (black), ARD, and extracellular linker (gray) between the two monophyletic groups. Color bars next to the zoomed alignment represent the groups (mauve: mammalian, orange: fish, black: hTRPV1-4).

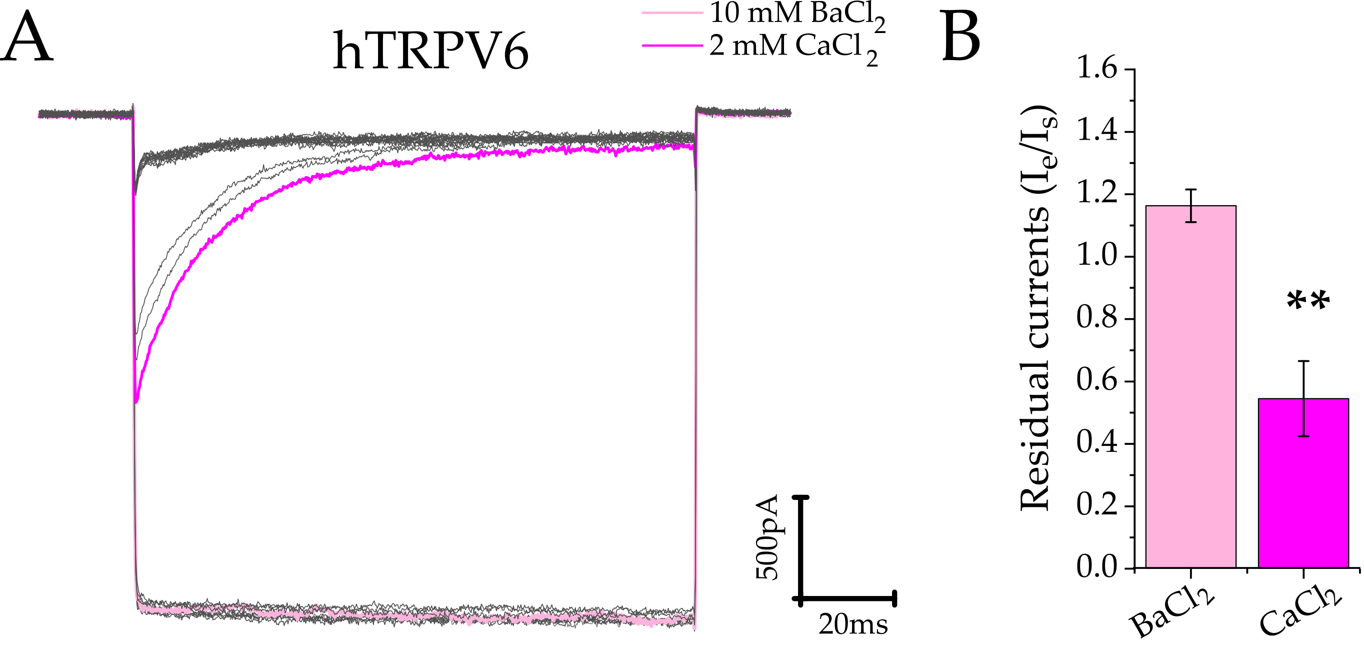

A
hTRPV6
10 mM BaCl2
2 mM CaCl2
500pA
20ms
B
Residual currents (Ie/Is)
1.6
1.4
1.2
1.0
0.8
0.6
0.4
0.2
0.0
**
BaCl2
CaCl2

SF2

Supplementary Materials Figure S2. Calcium-specificity of mammalian TRPV6 fast inactivation. (A) Representative current traces recorded from transiently transfected HEK-293T cells expressing wild-type (WT) hTRPV6 in response to repetitive 60ms -160mV pulses. Rapid current decay was observed after changing the extracellular solution from 10 mM Ba^{2+} (initial traces, light pink) to 2 mM Ca^{2+} (final traces, magenta). **(B)** Pooled data comparing the residual currents (defined as the ratio between the currents at the end (Ie) versus the beginning (Is) of the voltage pulse). Bars represent mean value; errors represent S.E.M, $^{**}p = 0.01$.

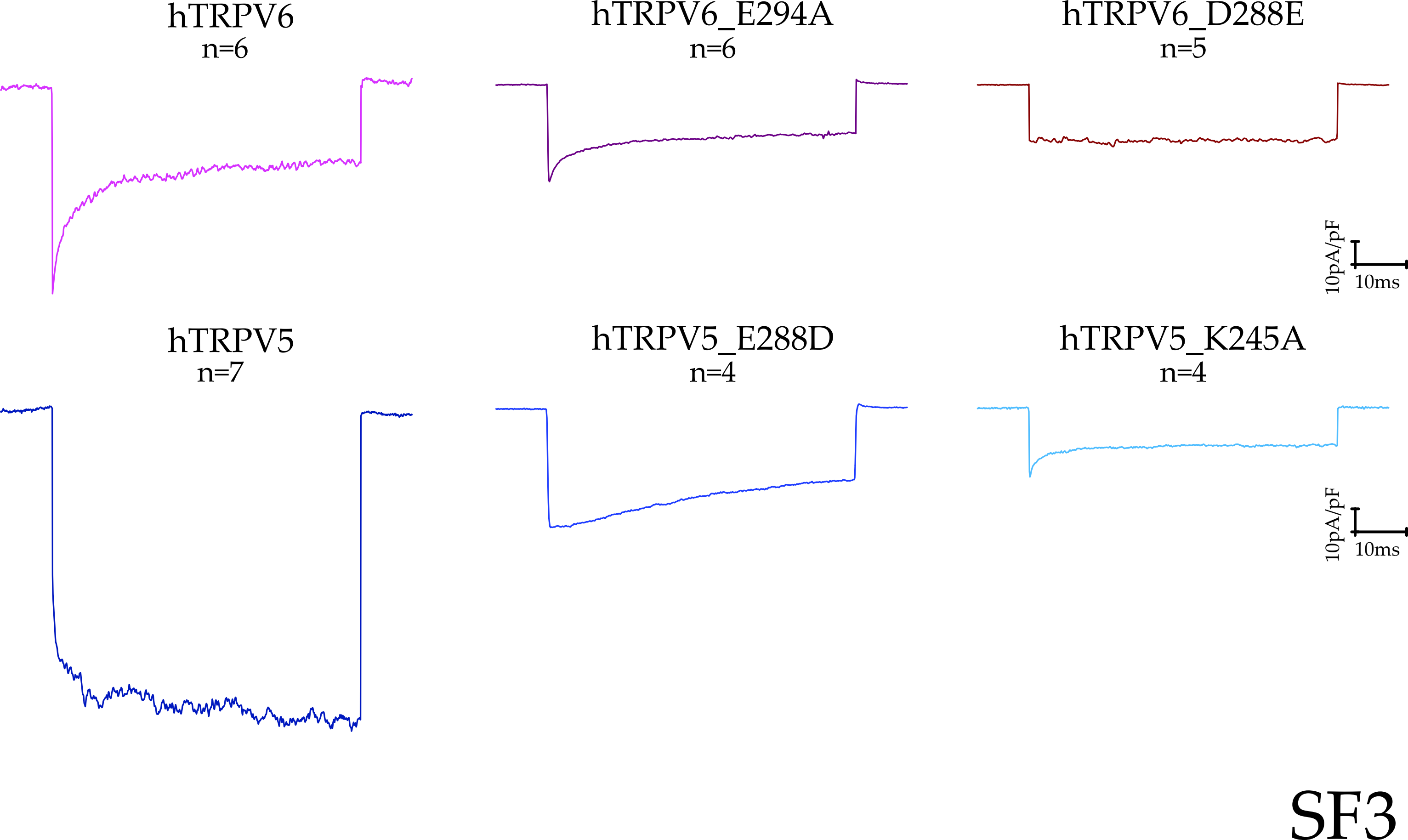
hTRPV6
n=6
hTRPV6_E294A
n=6
hTRPV6_D288E
n=5
10pA/pF
10ms
hTRPV5
n=7
hTRPV5_E288D
n=4
hTRPV5_K245A
n=4
10pA/pF
10ms
SF3

Supplementary Materials Figure S3. Representative current traces recorded from transiently transfected HEK-293T cells expressing WT hTRPV6, hTRPV6-E294A, hTRPV6-D288E, WT hTRPV5, hTRPV5 E288D, and hTRPV5-K245A, in response to a 60ms -160mV voltage pulse. Numbers represent n for each condition.

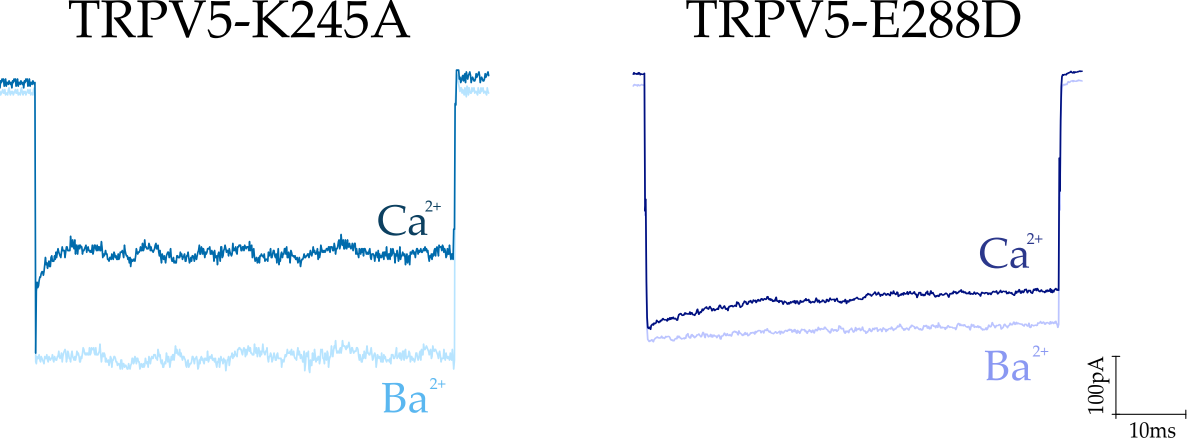

SF4

Supplementary Materials Figure S4. Calcium-specificity of the fast inactivation of calcium-selective TRP channels. (**A**) Representative current traces recorded from transiently transfected HEK-293T cells expressing hTRPV5-K245A and hTRPV5-E288D in response to repetitive 60ms -160mV pulses. Rapid current decay was observed after changing the extracellular solution from 10 mM Ba^{2+} (initial traces, light color) to 2 mM Ca^{2+} (final traces, dark color).

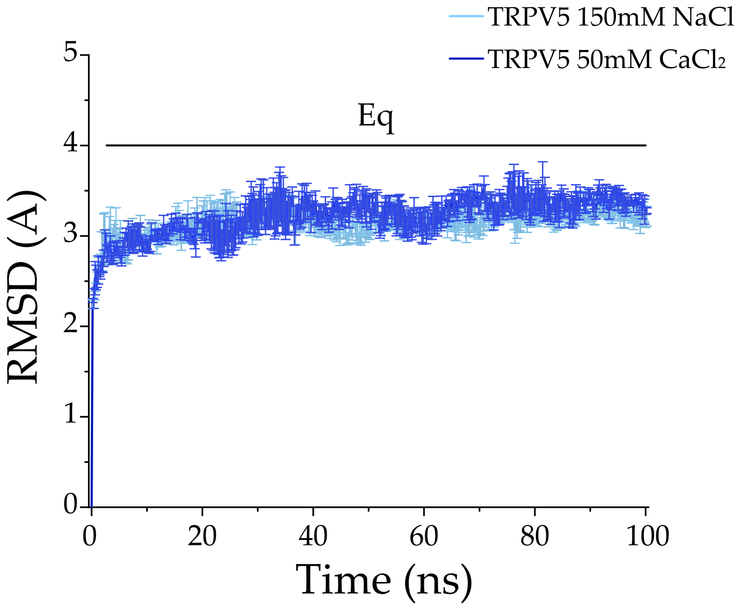

TRPV5 150mM NaCl
TRPV5 50mM CaCl2
Eq
RMSD (A)
Time (ns)
0
1
2
3
4
5
0
20
40
60
80
100

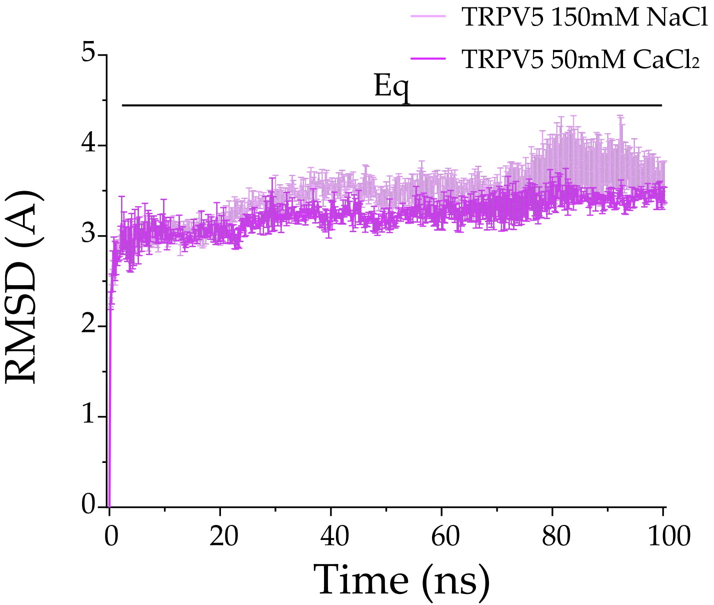

TRPV5 150mM NaCl
TRPV5 50mM CaCl2
Eq
RMSD (A)
Time (ns)
0
1
2
3
4
5
0
20
40
60
80
100

SF5

Supplementary Materials Figure S5. RMSD of TRPV5 and TRPV6 proteins during the 100 ns of molecular dynamic simulations in presence of 150 mM NaCl (light) or 50 mM CaCl2 (dark). The horizontal line represents the time considered equilibrium (Eq) and the timeframe used for the analysis.

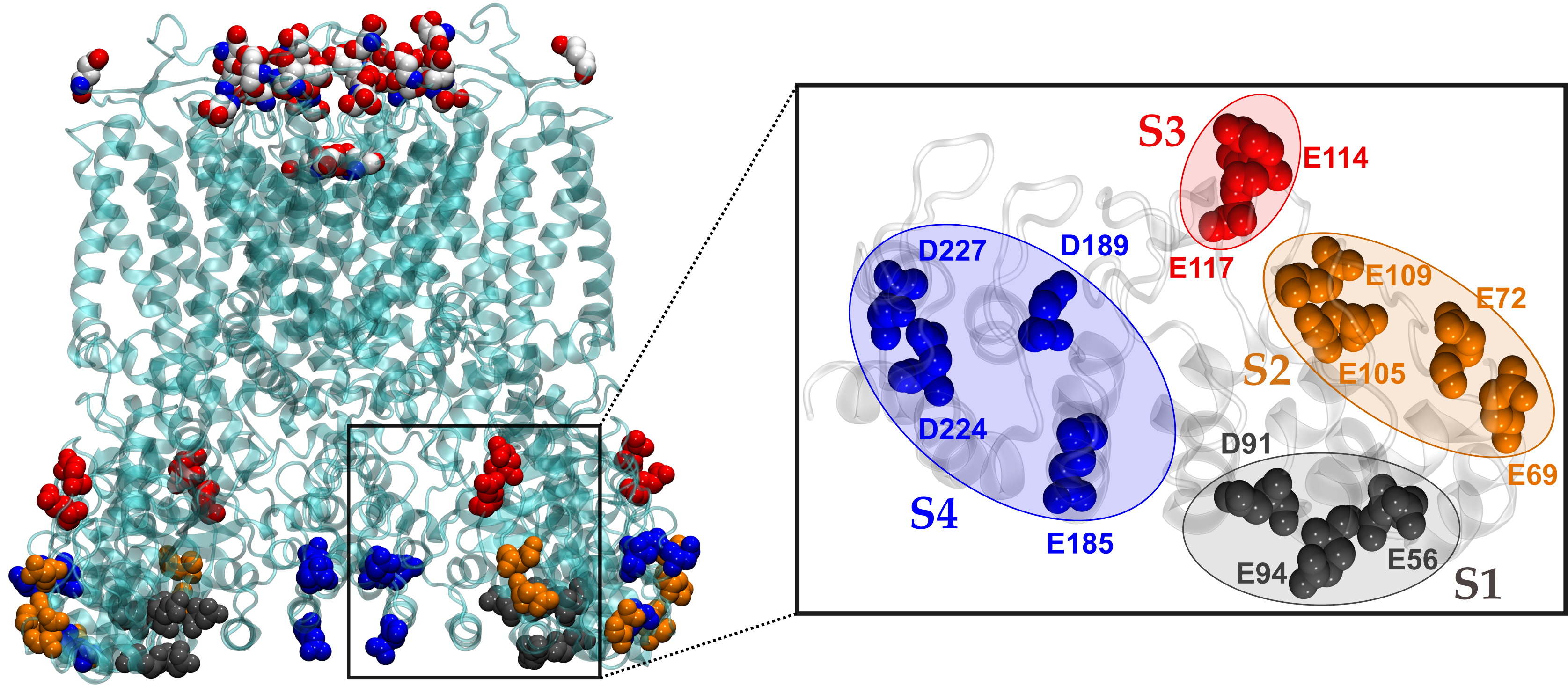
S3
E114
E117
D227
D189
E109
E72
S2
E105
D224
D91
E69
S4
E185
E94
E56
S1

SF6

Supplementary Materials Figure S6. Molecular dynamics simulation (MDS) predicts direct binding of Ca^{2+} ions to TRPV5-6 intracellular domains. The left panel shows a representative tetramer used for the MDS. Ca^{2+}-interacting residues located at the extracellular region of the channel are highlighted in the upper region, as well as residues forming the selectivity filter. Residues identified as putative intracellular Ca^{2+}-interacting sites are denoted by the color of the identified site. The right inset shows residues constituting the four putative Ca^{2+} interacting sites, denoted by the letter S, in one of the subunits. Site 1 in gray, Sie 2 in orange, Site 3 in red, and Site 4 in blue.

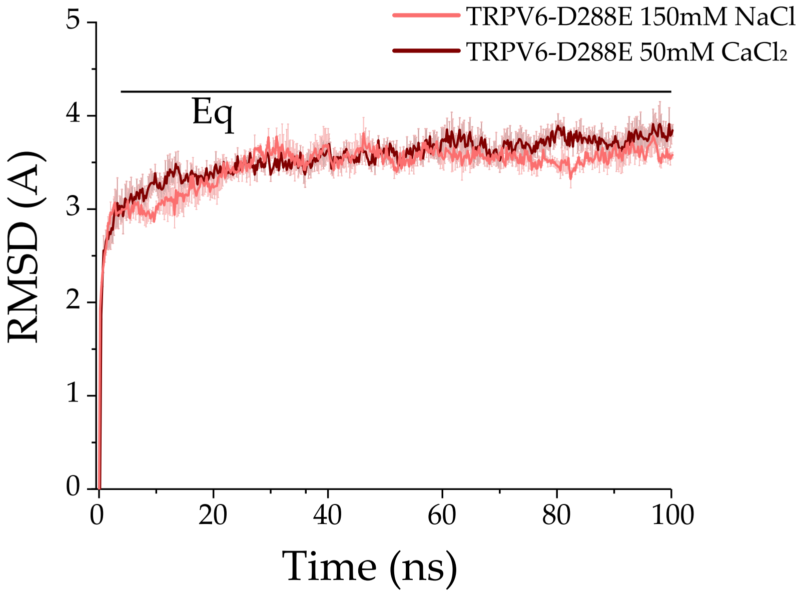

TRPV6-D288E 150mM NaCl
TRPV6-D288E 50mM CaCl2
Eq
RMSD (A)
Time (ns)
0
1
2
3
4
5
0
20
40
60
80
100

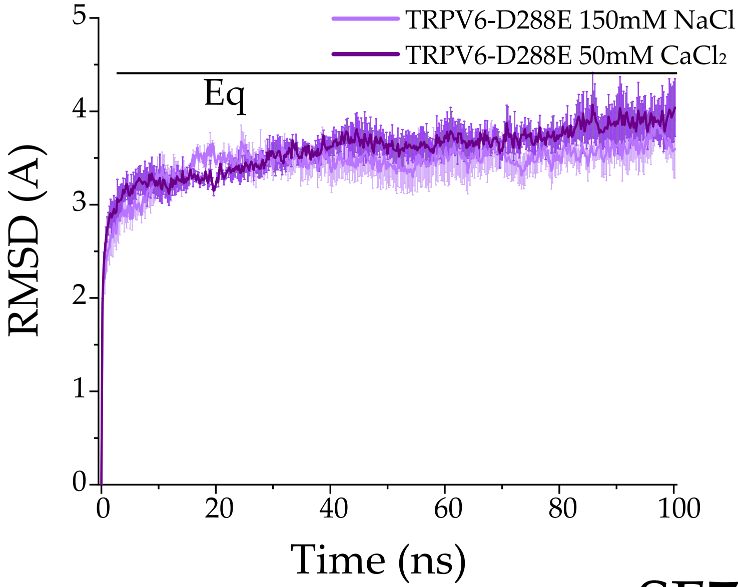

TRPV6-D288E 150mM NaCl
TRPV6-D288E 50mM CaCl2
Eq
RMSD (A)
Time (ns)
0
1
2
3
4
5
0
20
40
60
80
100

SF7

Supplementary Materials Figure S7. RMSD of TRPV6-D288E and TRPV6-E294A proteins during the 100 ns of molecular dynamic simulations in presence of 150 mM NaCl (light) or 50 mM CaCl2 (dark). The horizontal line represents the time considered equilibrium (Eq) and the timeframe used for the analysis

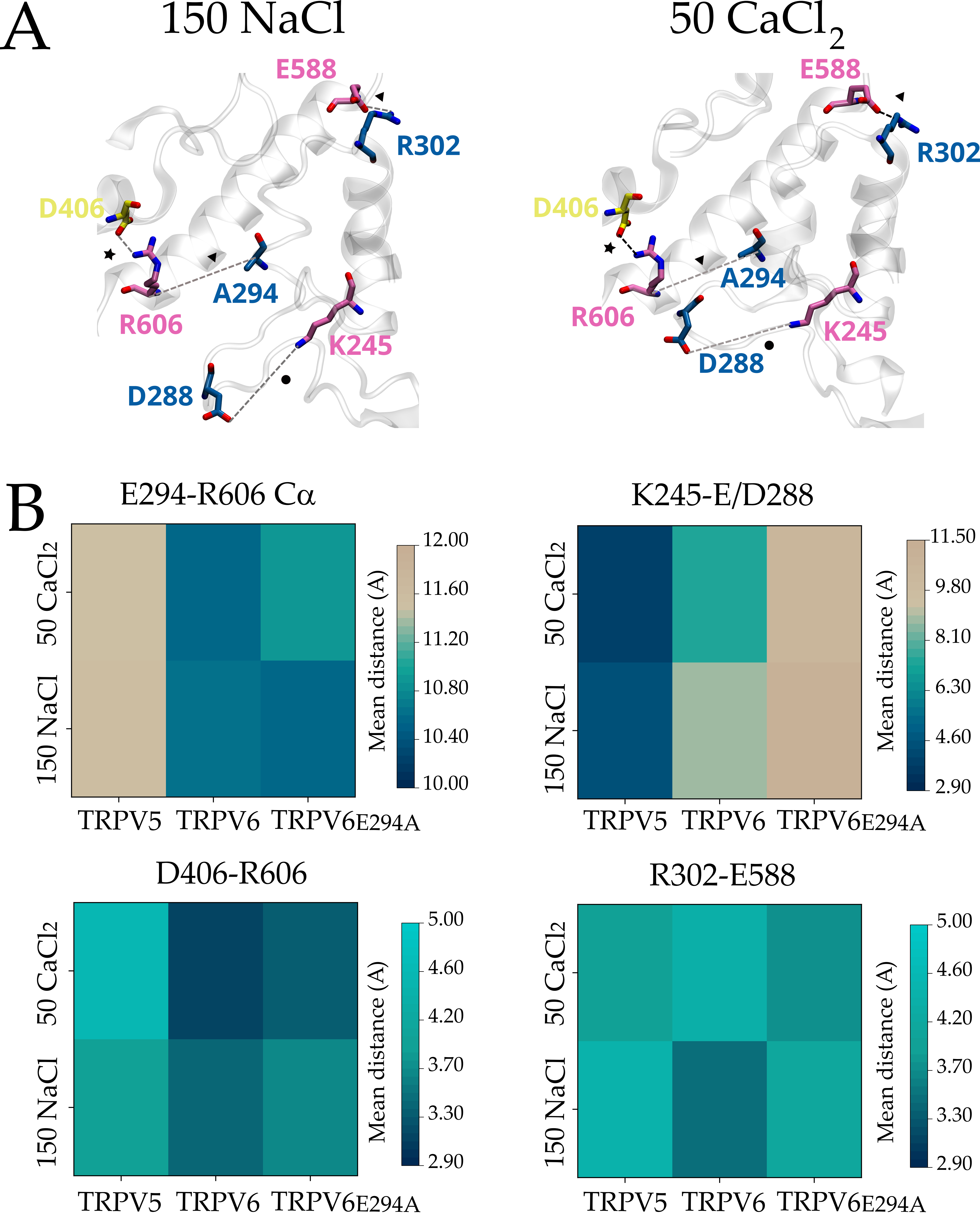
A
150 NaCl
50 CaCl2
E588
R302
D406
A294
R606
K245
D288
B
E294-R606 Cα
K245-E/D288
D406-R606
R302-E588
50 CaCl2
150 NaCl
TRPV5
TRPV6
TRPV6E294A
Mean distance (A)
12.00
11.60
11.20
10.80
10.40
10.00
11.50
9.80
8.10
6.30
4.60
2.90
5.00
4.60
4.20
3.70
3.30
2.90

SF8

Supplementary Materials Figure S8. Calcium ions trigger structural changes within the HLH/S2-S3 linker/ TDh inactivation motif in mutant TRPV6-E294A channel. **A**) Structural conformation of the three-dimensional HLH/S2-S3 linker/TDh inactivation motif of hTRPV6-E294A during 100ns of molecular dynamics simulations in presence of Na^+ or 50 Ca^{2+} ions. Residues of the HLH (blue), S2-S3 linker (yellow), TDh (mauve), and ARD (pink) contributing to interdomain interactions are highlighted, as well as the interactions (HLH/ARD: circle, HLH/TDh: triangle, S2-S3 linker: star). **B**) Heat maps representing the average distance between the pairs of residues in panel A during the molecular dynamics simulations in hTRPV5, hTRPV6, and hTRPV6-E294A. Bars on the right of each panel represent the color scale of the mean distance during the simulation time.
